# Loss of density-dependence and incomplete control by dominant breeders in a territorial species with density outbreaks

**DOI:** 10.1186/1472-6785-11-16

**Published:** 2011-07-04

**Authors:** Jana A Eccard, Ilmari Jokinen, Hannu Ylönen

**Affiliations:** 1 Animal Ecology, University of Potsdam, Maulbeerallee 1, 14469 Potsdam, Germany; 2 Department of Biological and Environmental Sciences, University of Jyväskylä, Finland; 3 Konnevesi Research Station, University of Jyväskylä, Finland

## Abstract

**Background:**

A territory as a prerequisite for breeding limits the maximum number of breeders in a given area, and thus lowers the proportion of breeders if population size increases. However, some territorially breeding animals can have dramatic density fluctuations and little is known about the change from density-dependent processes to density-independence of breeding during a population increase or an outbreak. We suggest that territoriality, breeding suppression and its break-down can be understood with an incomplete-control model, developed for social breeders and social suppression.

**Results:**

We studied density dependence in an *arvicoline *species, the bank vole, known as a territorial breeder with cyclic and non-cyclic density fluctuations and periodically high densities in different parts of its range. Our long-term data base from 38 experimental populations in large enclosures in boreal grassland confirms that breeding rates are density-regulated at moderate densities, probably by social suppression of subordinate potential breeders. We conducted an experiment, were we doubled and tripled this moderate density under otherwise the same conditions and measured space use, mortality, reproduction and faecal stress hormone levels (FGM) of adult females. We found that mortality did not differ among the densities, but the regulation of the breeding rate broke down: at double and triple densities all females were breeding, while at the low density the breeding rate was regulated as observed before. Spatial overlap among females increased with density, while a minimum territory size was maintained. Mean stress hormone levels were higher in double and triple densities than at moderate density.

**Conclusions:**

At low and moderate densities, breeding suppression by the dominant breeders, But above a density-threshold (similar to a *competition point*), the dominance of breeders could not be sustained (*incomplete control*). In our experiment, this point was reached after territories could not shrink any further, while the number of intruders continued to increase with increasing density. Probably suppression becomes too costly for the dominants, and increasing number of other breeders reduces the effectiveness of threats. In wild populations, crossing this threshold would allow for a rapid density increase or population outbreaks, enabling territorial species to escape density-dependency.

## Background

### Territoriality and breeding suppression

**Figure 1:**
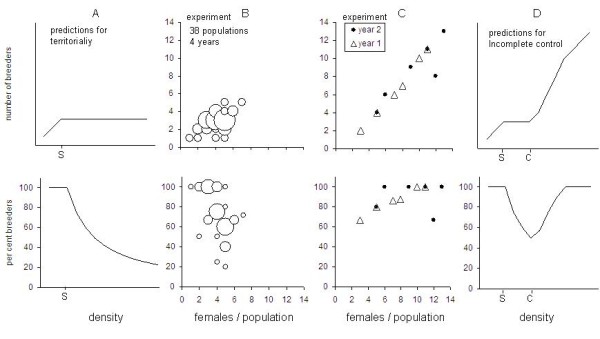
**A: Conceptual model of density dependence of the number of breeders and the proportion of breeders in territorial species**. (S) Saturation point where all territories are occupied and breeding suppression begins. B: Empirical evidence for density dependence in 38 enclosed experimental bank vole populations at moderate densities [5,25-28,41]. The size of the symbol represents the number of population with the same value. C: Results of the experiment (this paper) extending the density range of enclosed bank vole populations to medium and high densities. Each symbol represents one population. D: Conceptual model of a territorial species breeding suppression (control) at moderate densities (from density S to density C) and release of suppression (incomplete control) at higher densities (density > C, C can be compared with the 'competition point' in eusocial insects).

Territoriality in a breeding species includes a defended area that is considered to have resources enhancing reproductive success compared to that of conspecifics [1], or that allows to defend offspring against conspecifics [2]. Mean territory size depends on resources within the territory, which may be food or shelter for example for female, lactating mammals, and may vary between years or seasons the number of available territories is the upper limit of the number of breeders on a given area [3]. The breeding activities of non-territory holders are suppressed, thus, territoriality is preventing excess animals from breeding [4]. Territoriality therefore causes reproductive skew between territorial breeders and non-territorial non-breeders. Accordingly, if population numbers rise, the number of breeders remains stable and the proportion of breeders drops [5] while the number of offspring, i.e. the recruitment of a population, remains stable (for a conceptual illustration see Figure 1A). Territoriality can therefore act as a density regulation mechanism [6] and consequently, truly territorial species should lack the ability to produce density outbreaks.

Breeding suppression of others is costly for the breeders, since it involves aggressive interactions towards the non-breeders or production of suppression pheromones [e. g. [7]]. Furthermore, the fitness value of the offspring decreases with density increase, as the probability of offspring's genetic contribution to the peak density decreases. Therefore breeding suppression in increasing density not only increases in costs, but also loses benefits. For cooperative breeding animals, the costs and benefits of suppression both for the dominant and subordinated have been modelled in tug-of-war, incomplete-control [8] or concession models [9]. In social insects a maximum of suppressible workers was found at the 'competition point' [10], above which the suppression of breeding of workers by the queen fails [11].

### Population cycles and outbreaks

Density outbreaks and regularly occurring high densities are described for many species and their causalities are constantly debated. Regular cyclic patterns are best know in snow-shoe hares in Yukon, Canada [e. g. [12]] and in northern-boreal vole communities in Fennoscandia [e. g. [13]] or in Hokkaido, Japan [14]. Famous examples of non-periodic outbreaks in rodents include those of European house mouse in Australian grain-growing areas [e. g. [15]] and outbreaks of common vole in Central-European agricultural areas [e. g. [16]], but see [17].

Especially the cyclic fluctuations of small mammal densities have received much attention and controversial explanations [for review e.g. [18]]. Density-dependent processes with little or short time lag tend to stabilize population size and density-independent processes will destabilise it [19]. Spacing behaviour, territoriality and breeding suppression should act as self-regulating processes [20]. However, some of the arvicoline species exhibiting regular high population densities, i.e. all voles of the genus *Myodes *are described as territorial, with females defending an exclusive territory as a prerequisite for breeding [21,22]. Theoretically this should mean that the population cannot increase more as local territory density has been saturated (Figure 1A).

Reports on *Myodes*' spacing behaviour and breeding support predictions above made for territoriality: the number of breeders is limited by social suppression in wild populations [23,24]. Stronger females suppress the maturation of weaker females after winter [5,25] adults suppress the maturation of young females in late summer, both in enclosed [26-28] and in wild populations [29]. Breeding suppression in social breeders had also been discussed as an adaptive delay of breeding by subordinates, a restraint of subordinates foregoing breeding in expectation that conditions could improve in the future [30]. In small rodents, however, individual lifetime is very short and predation risk is extremely high [31], and, in our study area, breeding season lasts only few months. There is only a very small chance ever for an individual to ever reach this future, we therefore regard suppression as a constraint, not as an adaptive restraint [32].

Exclusive female territoriality thus prevents surplus adult, mature females from breeding if breeding density is saturated [27,28]. In contradiction to this, *Myodes *voles are among those arviclone rodents that produce cyclic and non-cyclic density outbreaks in part of their range [33,34], a phenomen that should be precluded by their territoriality [35].

### Aims

There is an apparent contradiction between density regulation by means of breeding territoriality and suppression of maturation in subordinate individuals on one hand, and the nevertheless occurring density peaks or outbreaks in the same species. To investigate this we conducted an experiment where densities, known to produce breeding suppression, were doubled and tripled. We measured effects of density on life history, behaviour and physiology of bank vole females. We combine earlier evidence from breeding suppression in low densities (Figure 1B) and the results of this study (Figure 1C) to a model that allows both breeding suppression and density outbreaks, using an incomplete-control argument (Figure 1D).

To understand what happens at the 'competition point', i.e. the point where breeding suppression apparently fails (density C in Figure 1D), we monitored spatial behaviour, reproduction and physiology of the individual females. Spacing behaviour was inferred from live-trapping and was used to describe potential interaction among females. We further measured faecal glucocorticoid metabolites (FGM) levels as a proxy for density-induced corticosterone levels, the major stress hormone in most rodents. Stress responses associated with high density were hypothesized to play a major role in population regulation of wild rodent with regular density fluctuations [e. g. [36-38]], but this has also been disputed [39,40]. With this multivariate approach we tried to assess the mechanisms regulating breeding density in a territorial breeder, and how regulation fails.

## Methods

### Breeding under moderate densities

We have studied artificial bank vole populations in large outdoor enclosures (0.25ha) in Central Finland over many years. Voles were clearly territorial and density-limited in the enclosed system we used, similar to what is reported from wild populations [21,23]. In the experimental populations, 3-4 females were breeding simultaneously, and we regularly started our experiments with populations of 5 females to buffer predation and natural mortality. To evaluate if here that breeding suppression is evident also in our enclosed system, we compiled a data base, consisting of 38 un-manipulated, experimental populations from different studies in the years 1997-2000. These populations, because they served as controls for various experimental treatments, did not undergo any kind of experimental treatment during the respective study periods.

In all populations of these earlier studies, 5 laboratory born females, descending from a colony of wild captured bank voles from the region were introduced to the enclosures. This resulted in 4.1 ± 1.2 (mean ± SD) potential breeders. In those of the studies that were conducted during summer (n = 27 populations) the females were adult and multiparous [5,27,28] but not pregnant at the release to the enclosures. Three males were introduced 2-3 days after the females and remained in the enclosures for at least 1 week, but often as long as the females, depending on study design. Breeding and survival was monitored for one pregnancy cycle (= 3 weeks). In those of the studies which were carried over winter to monitor the onset of breeding and the first reproduction in spring (n = 11 populations) [25,41] young immature females and males were introduced into the enclosures in October. Breeding rates of survivors were monitored in spring.

#### An experiment with increasing densities

All our earlier studies covered a density range of 8-20 females per hectare (i.e. 2-5 females/enclosure), but we had no information on breeding suppression, interaction and physiological responses in higher densities. We therefore conducted an experiment during the summers 2001 and 2002 using 12 enclosed experimental populations. We repeated, doubled and tripled the above described, initial density of 5 adult females. We used two enclosures for each density treatment in each of the summers, resulting in four replicate populations for 5, 10 and 15 females per enclosures, resembling 20, 40 and 60 females/hectare, which are comparatively high densities for potentially breeding females in open field populations [25,42]. Females were transferred to the enclosures at day 0 of the experiment (see experimental schedule, Table 1). Three, 6 and 9 males, respectively, were introduced in each population after 4 days of female habituation to ensure comparable operational sex ratios, and to produce synchronized pregnancies. Experimental voles were offspring from a permanent laboratory colony. Most were born during the preceding autumn and had over-wintered in the laboratory, as the majority of breeding females in early and mid summer in wild populations. All females had successfully bred in the laboratory and weaned litters. Females were not pregnant at the beginning of the experiment. At transfer to the enclosures, experimental populations consisted of non-related females of similar age composition among populations. Animals were ear-tagged for individual identification. They were removed from the enclosures shortly before giving birth (20 days ± 2 days length of pregnancy, compare Table 1) and were returned to single cages. We measured survival rates, pregnancy rates, birth dates of litters and litter sizes.

**Table 1:** Experimental schedule for 12 populations in two summers.

experimental day	experimental protocol
-5 to -3*	faecal samples of females from single cages
0	transfer of females to field enclosures
2-4*	morning trapping, faecal samples of females
4	transfer of males to field enclosures
12*	morning trapping, faecal samples of females after 1^st ^week
17*	morning trapping, faecal samples of females after 2^nd ^week
18-22	live-trapping (day 18 evening to day 22, morning)
23*	morning trapping, faecal samples of females after 3^rd ^week
23-25	trapping and transfer of animals to laboratory
25-30	monitoring birth of litters in laboratory

FGM sampling (*) was conducted only during the 1st year

Towards the end of the field period (Table 1, day 18-22 after release to the field) we conducted live-trapping to estimate space use. Traps were set in the evening of the 18^th ^experimental day, controlled 3-times per day, and opened on the 22^nd ^day in the morning. The trap stations in the enclosures were permanently installed, well visited, and attractive sites for the animals with regular provisioning of bait during trapping. We therefore assumed that captures of several animals at the same location (not necessarily at the same time) were indicative for potential interaction among these individuals. Since we had no information on aggressive interactions, we used the number of exclusive trap locations, i.e. locations where exclusively only one female was captured, as an indicator of exclusive space without interaction. For territorially breeding bank vole females, exclusive space is an important prerequisite for breeding [43].

During the first summer, physiological estimators of density stress were obtained by measuring faecal glucocorticoid metabolites (FGM), a non-invasive and non-terminal measurement. We followed a sampling, extraction and analysis protocol of Harper and Ausstad [44] and slightly modified it as described in detail in our paper [45]. In short, samples were boiled in ethanol and assayed with a commercial kit (ImmuChem Double Antibody Corticosterone ^125^I RIA, MP Biomedicals, CA, USA) intended for the analysis of plasma and extracted urinary samples of rats and mice. Faeces of bank vole females was collected once a week (up to four measurements per female, Table 1) and sampled during morning hours to minimize variation due to daily FGM fluctuations [46]. Hormone metabolites from stressful events show in the faeces of rodents with similar body size and diet after 4-6 h [46,47]. In order to sample only the pre-trapping stress hormone levels, we collected samples at latest 3 h after setting the trap, i.e. animals had been captured less than 3 h ago. Between the last space trapping (open traps 22^nd ^experimental day, morning) and the third FGM sampling (set traps 23^rd ^day, morning) elapsed a period of 24 h without trapping or handling of animals.

Comparison of FGM concentration sampled from the individuals before the onset of the field phase (still in single cages) showed, that the method a) reflects stressful events and b) reflects individual variation. a) Shortly after release to the enclosures, when the environment physical and social environment was unknown to the cage-bred females, FGM concentrations were on average 5 fold of the individual values sampled in the laboratory (laboratory: 1.4 ± 1.6, after release: 5.9 ± 8.3 ng FGM/mg faeces, paired t-test, t = -3.5, n = 37, p = 0.001). b) Individuals with relatively high FGM levels in the laboratory had also relative high FGM levels shortly after release (Pearson's rho = 0.410, n = 38, p = 0.011).

Density effects in breeding performance, spacing behaviour and physiology were analysed on the population level, using enclosure rates or enclosure means. This was done to avoid pseudo replication [48]. We used the number of surviving females (as indicated during the space trapping) instead of the initial density treatment, because treatments later in the experiment overlapped: 3-6 females in the low density treatment, 7-11 in the double, and 10-13 in the triple density treatment. The use of a gradient instead of the initial treatment seemed therefore more appropriate. Effects of density and year were investigated with an analysis of covariance (ANCOVA, density as covariate, year as factor). Since neither year nor an interaction of year and density turned out to be significant in any of the tested variables, we here present the effects of density as regression models. We tested for both linear models (y = ax+b) and/or an inverse models (y = a/x+b). For significant regression models we give coefficients and constants in Table 2. The constant b in the inverse model indicates a threshold value for indefinitely high densities. All statistics were computed with SPSS (Version 17, SPSS Inc, Chicago Illinois).

**Table 2:** Regression models for the effect of density (number of females per enclosure) on variables of reproduction, space use and physiology in enclosure experiments on bank voles.

study	variable	model	R2	n	F	p	coefficient a	constant b
**moderate densities (4-20 bank vole females/ha)**								
	breeding females	inverse	0.230	38	12.1	0.001	-3.37	3.78
	breeding females	linear	0.265	38	14.3	0.001	0.43	1.1

**moderate to high densities (12-42 bank vole females/ha)**								
	breeding females	linear	0.817	12	50.2	< 0.001	0.83	-2.33
	breeding females	inverse	0.674	12	23.7	0.001	-70.6	14.2
	mean litter size	linear	< 0.01	12	< 0.01	0.931		
	
	mean home range	inverse	0.789	12	37.5	< 0.001	2420	364
	interaction (nr. of females)	linear	0.653	12	18.9	0.001	0.45	0.58
	captures/animal	linear	0.3	12	4.2	0.065	-0.21	8.7
	
	Log(FGM 1^st^week	inverse	0.035	6	0.1	0.723		
	Log(FGM 2^nd^week)	inverse	0.8	6	16	0.016	-4.6	2.9
	Log(FGM 3^rd ^week)	inverse	0.562	6	5.1	0.086		

We tested linear model (y = ax+b) and/or an inverse models (y = a/x+b), the latter indicating a threshold value (constant b) for high densities. Analyses were based on populations (rates or enclosure means). 38 Populations at moderate densities were investigated over 4 years in different earlier experiments (see text), 12 populations in moderate to high densities were investigated during the 2-year experiment reported here.

The date reported here stems from several studies, which all were conducted under approval of the Committee for Animal Experimentation at the University of Jyväskylä. In all studies the set-up was such, that it caused no harm to the individuals of the experimental populations. The sampling for measurements of stress levels was carried out non-invasively by monitoring hormones in the faeces. The permission number for the last experiment was 34/31.5.2004, provided by the ethical committee named above.

## Results

### Survival and breeding performance in moderate densities (Figure 1B)

In 68% of the observed populations breeding suppression of at least one of the surviving females was observed, in 40% of the populations two or more females were not able to breed (Figure 1B). Of a mean number of 4.1 ± 1.2 (mean ± SD) females per population alive after three weeks, the mean number of breeders was 2.8 ± 1.0 per population. The inverse model predicted 3.8 breeding females as a theoretical threshold value for infinitive numbers of females (Table 2).

### Survival and breeding performance along increasing densities (Figure 1 C, Table 2)

Survival of females did not differ between enclosures of the three density treatments (F _2,8 _= 2.6, p = 0.132) but differed between years (F _1,8 _= 11.8, p = 0.009, non-significant interaction term removed) with a survival of 68 ± 16% (mean ± SD) of females per enclosure in 2001 and 92 ± 10% in 2002, respectively.

In initial moderate densities 67-100% of females were breeding (mean: 82%), in initially double densities 86-100% (mean 93%) and in triple densities all females in three populations and in the fourth one 67% of the females were breeding (Figure 1 C). The increase can be explained by density with a steep slope of 0.89 (linear) or a threshold value of 14 females breeding (inverse, Table 2). Mean litter size was not explained by density (Table 2).

### Space use (Figure 2A Table 2) in moderate to high densities

Mean home-range size decreased with density towards a threshold value of 364 sqm (inverse model). Mean number of females overlapping each other home-ranges increased linearly, indicating that the exclusive space within the home-range decreased linearly without reaching a threshold.

### Physiological measures (Figure 2B)

Enclosure means of FGM concentrations were analysed with repeated measures analysis of variance (rmANOVA) using the mixed model procedure in SPSS. Enclosures were used as subjects, sampling weeks as repeats (1^st^, 2^nd^, 3^rd ^week), and females per enclosure during the space trapping as the covariate. Different covariance structure of repeats were modelled and the best (lowest) AIC [49] was obtained with an ante-dependence structure. This covariance structure is applicable to a repeated measurement design, in which the measurements are ordered in time and the present measurement depends only on the immediate antecedent measurements [50].

FGM concentrations were increasing with density and differed between sampling periods (fixed effects: covariate density F (1, 5.5) = 40.6, p = 0.001; factor sampling week F (2, 6.2) = 10.8, p = 0.01, ns interaction removed). Measurements were similar between 1st and 3rd week (paired t = 0.3, p = 0.795) but measurements in the 2^nd ^week differed from 3^rd ^(paired t = 4.1, p = 0.010) and tended to differ from 1^st ^week (paired t = 2.5, p = 0.053). Correlations to female density were due to an increase with density in the 2^nd ^week, and a tendency to increase with density during the 3^rd ^week. Measurements during the first week were not related to density (Table 2), but probably still elevated as a consequence of the new physical and social environment for females born in captivity and experiencing only laboratory life before.

## Discussion

### Territoriality and breeding suppression

Under moderate densities breeding suppression worked as predicted and similarly like observed in wild populations of bank voles [21,51]. At very low densities, all females were able to breed (Figure 1B) but after the saturation of breeding territories the breeding rate dropped, and breeding of excess animals was suppressed (Point S in Figure 1A, parameterised for our empirical system in Figure 1B at ~3 females per enclosure). However, when comparing enclosures at higher densities, breeding suppression was present only within a limited density range (between density S and density C in Figure 1D, parameterised for our system at 3-6 females per enclosure). Above this density range in all but one population, each single female was able to breed.

Female spacing behaviour and breeding suppression has been modelled as a classical *tragedy-of-the-commons *problem [52]. While breeding suppression benefits the breeders, as their genetic share in the population increases, it does not benefit the suppressed individuals. In territorial short-lived animals, each individual should gain a territory and suppress the breeding of weaker, non-territory holders to increase its genetic share of future generations [1]. At the same time, each non-breeding individual should challenge its status constantly. As long as numbers are low, territorial females can suppress breeding of others through aggressive interactions towards the non-breeders [4]. With higher densities in our study, the number of overlapping females rose linearly (Table 2). Females cannot indefinitely increase their allocation of energy and time into aggressiveness to suppress breeding of others, and therefore the number of female's aggressive acts towards each non-territory holders probably decreased until the aggressions are not sufficiently frequent to suppress breeding. Similar as described for breeding suppression in social breeders where animals are believed to be confronted costs of suppression which increase with group size [8-11], we suggest an incomplete control mechanism for the loss of density dependence and breeding suppression in territorial, non-social breeders.

**Figure 2:**
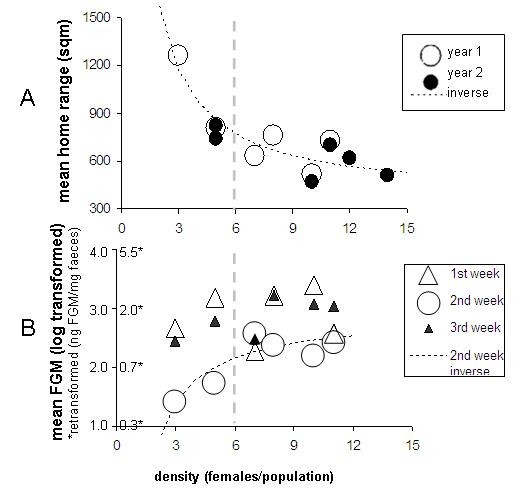
**Behaviour and physiology in enclosed experimental bank vole populations over different densities**. Mean home range size of females (A), mean faecal glucocoricoid metabolites (FGM) measured at weekly intervals (only one year, B). Each symbol represents the mean value for all females of one population. Thin dashed lines show predictions (inverse models), fat, dashed line: suggested threshold density for incomplete control

The mechanism of breeding suppression changing to incomplete control would be supported both by our results on space use and FGMs. Both variables ceased to be density-dependent at higher densities and were best explained by an inverse-function density-dependent model with a saturation value of +- 6 females/population (Figure 2A and 2B). Above this value, spacing behaviour of females shifts from a density-dependent process with the consequence of breeding suppression to a density-independent process without breeding suppression. In our conceptual model (Figure 1D) the threshold represent the density C, where social suppression is released and this may become possible trough the incomplete control mechanism. Apparently, the size of the females home range cannot shrink any further since it has to secure food resources necessary for breeding [53] or to ensure the shortest distance between nest sites to protect the vulnerable pups in the nest against possible infanticidal intruders [2]. At this point the FGM levels do not rise any further, either because the increase of home range overlap is stressfull *per se*, independent of the number of intruders, or because the response has reached physiological limits unknown to us.

Although we documented elevated levels of faecal stress hormone titres in higher densities (Figure 2B), we found no negative effects on physiological performance of females, not on survival rates, pregnancy rates or litter sizes. Possibly the earlier proposed density-dependent stress response of breeding and survival [37,38] is a long-term process and three weeks of high density as in our experiment were too short to affect females' reproduction or mortality. Our results do support though, that elevated densities can cause elevated stress levels in the individual, and we may speculate that persistent exposure to stress could have caused breeding reductions or survival reductions.

### Population cycles and outbreaks

Density outbreaks or exceptional high densities are described for many species normally exhibiting low or moderate densities for extended periods over several years or even decades [54]. Even under favourable environmental conditions some species represent a social system based on breeding territoriality in females. Only a fairly stable number of females per unit of favourable breeding habitats can produce offspring at a time independent of total population density [55]. This means that in the population there is a great proportion of potential breeders waiting for an opportunity to reproduce [e. g.[29]]. These, however, are short-lived with low probabilities to survive to the next breeding season if not able to breed in the summer of birth, like in our study species the bank vole [32].

Density-dependent processes tend to stabilise population densities, while density-independent processes tend to destabilise them [19]. In microtine cycles, population-intrinsic mechanisms of still unknown nature may be essential for the cycles to occur, but self-regulation must "not be too strong, though" [20]. Our experiment on bank voles was too short to investigate cyclic population dynamics among years. However we were able to observe a failure of territorial breeding suppression of bank voles at higher experimental densities. Density increase did not continuously affect reproduction and breeding, we rather found a threshold above which the density-dependence apparently broke down. Our findings may shed light on the spatial and interactive mechanisms explaining unexpected outbreaks of species, which are territorial at low densities. Further, the concept of incomplete-control mechanisms developed for understanding the evolution of cooperative breeding may extend to breeding territoriality and population biology.

## Conclusions

Territoriality and breeding suppression should limit the breeding density and reproductive output of a species. However, there are territorial species with, under a territorial breeding system, inexplicable density outbreaks. Here we have shown in an experiment with bank voles, a territorial breeder at low densities, that above a density-threshold the dominance of breeders could not be sustained. This observation can be explained using a incomplete-control framework, originally developed for social breeders, where the density-threshold can be interpreted as the competition point. In our experiment, this point was reached after territories could not shrink any further, but the number of intruders continued to increase. Probably suppression becomes too costly for the dominants, and increasing number of other breeders reduces the effectiveness of threats. In wild populations, crossing this threshold would allow for a rapid density increase or population outbreaks, enabling territorial species to escape density-dependency.

## Authors' contributions

JAE conceived the study and design, worked in the field, conducted the statistical analysis and drafted the manuscript. HY participated in study design, worked in the field and coordinated the field work. IJ validated and coordinated the FGM Analysis. All authors corrected and improved the manuscript.
